# MicroRNA-Based Prophylaxis in a Mouse Model of Cirrhosis and Liver Cancer

**DOI:** 10.1016/j.omtn.2018.11.018

**Published:** 2018-12-06

**Authors:** Elisa Callegari, Marco Domenicali, Ram Charan Shankaraiah, Lucilla D’Abundo, Paola Guerriero, Ferdinando Giannone, Maurizio Baldassarre, Cristian Bassi, Bahaeldin K. Elamin, Barbara Zagatti, Manuela Ferracin, Francesca Fornari, Giuseppe Altavilla, Stella Blandamura, Enrico Maria Silini, Laura Gramantieri, Silvia Sabbioni, Massimo Negrini

**Affiliations:** 1 Department of Morphology, Surgery and Experimental Medicine, University of Ferrara, 44121 Ferrara, Italy; 2 Department of Medical and Surgical Sciences, Alma Mater Studiorum University of Bologna, 40138 Bologna, Italy; 3 Center for Applied Biomedical Research, St. Orsola-Malpighi University Hospital, 40138 Bologna, Italy; 4 Department of Life Sciences and Biotechnologies, University of Ferrara, 44121 Ferrara, Italy; 5 Department of Basic Sciences, College of Medicine, University of Bisha, 61922 Bisha, Saudi Arabia; 6 Department of Medical Microbiology, Faculty of Medical Laboratory Sciences, University of Khartoum, 11115 Khartoum, Sudan; 7 Department of Experimental, Diagnostic and Specialty Medicine (DIMES), University of Bologna, 40126 Bologna, Italy; 8 Department of Medicine DIMED, University of Padova, 35121 Padova, Italy; 9 Section of Anatomy and Pathology, University Hospital of Parma, 43121 Parma, Italy

**Keywords:** hepatocellular carcinoma, HCC, cirrhosis, carbon tetrachloride, CCl_4_, transgenic animals, microRNAs, prophylaxis

## Abstract

Most hepatocellular carcinomas (HCCs) arise in the context of chronic liver disease and/or cirrhosis. Thus, chemoprevention in individuals at risk represents an important but yet unproven approach. In this study, we investigated the ability of microRNA (miRNA)-based molecules to prevent liver cancer development in a cirrhotic model. To this end, we developed a mouse model able to recapitulate the natural progression from fibrosis to HCC, and then we tested the prophylactic activity of an miRNA-based approach in the model. The experiments were carried out in the TG221 transgenic mouse, characterized by the overexpression of miR-221 in the liver and predisposed to the development of liver tumors. TG221 as well as wild-type mice were exposed to the hepatotoxin carbon tetrachloride (CCl_4_) to induce chronic liver damage. All mice developed liver cirrhosis, but only TG221 mice developed nodular lesions in 100% of cases within 6 months of age. The spectrum of lesions ranged from dysplastic foci to carcinomas. To investigate miRNA-based prophylactic approaches, anti-miR-221 oligonucleotides or miR-199a-3p mimics were administered to TG221 CCl_4_-treated mice. Compared to control animals, a significant reduction in number, size, and, most significantly, malignant phenotype of liver nodules was observed, thus demonstrating an important prophylactic action of miRNA-based molecules. In summary, in this article, we not only report a simple model of liver cancer in a cirrhotic background but also provide evidence for a potential miRNA-based approach to reduce the risk of HCC development.

## Introduction

Hepatocellular carcinoma (HCC) is the second leading cause of cancer-related death worldwide.^1^ Liver cirrhosis is the main risk factor for HCC, as approximately 80% of tumors develop in individuals with advanced liver fibrosis or cirrhosis.^2^ Viral hepatitis B and C and alcohol abuse are the main causes of cirrhosis and liver cancer; however, non-alcoholic fatty liver disease and steatohepatitis are emerging contributing factors for this disease in developed countries.^3^, ^4^

Therapeutic approaches in patients with HCC mostly depend on liver function and tumor extension, and they include surgery, percutaneous ablation, chemoembolization, and radioembolization.^5^ Sorafenib, the only approved systemic therapy for patients with unresectable advanced-stage HCC, provides only a small improvement in patients’ survival.^6^, ^7^ Additional targeting agents have been tested, alone or in combination with sorafenib, to improve HCC therapies with limited survival benefits.^8^, ^9^, ^10^ Thus, new therapeutic approaches are certainly needed; but, given the existence of a well-defined at-risk population, chemoprevention represents another important but yet unproven approach.

The pivotal role of microRNAs (miRNAs) in cancer^11^ opened new avenues for clinical applications. Indeed, for their ability to regulate oncogenes and tumor suppressor genes, miRNA-based therapies have emerged as promising anti-cancer strategies.^12^ In HCC, miR-221 is overexpressed in approximately 70%–80% of human cases, and it is known to downregulate the expression of multiple gene targets relevant for cancer, such as the cyclin-dependent kinase inhibitors p27 and p57, the phosphatase and tensin homolog (PTEN), or the BH3-only pro-apoptotic protein BMF.^13^, ^14^, ^15^ We have validated the importance of miR-221 in liver tumorigenesis by developing the TG221 transgenic mouse, which is characterized by an inappropriate overexpression of miR-221 in the liver, emergence of spontaneous nodular liver lesions in approximately 50% of male mice at 12 months of age, and accelerated development of HCC upon treatment with diethylnitrosamine (DEN).^16^ Another important miRNA, miR-199a-3p, is downregulated in virtually all HCCs,^17^ and it is involved in the control of several cancer-associated genes, such as mechanistic target of rapamycin (mTOR), the hepatocyte growth factor receptor MET, the kinase p21-activated kinase 4 (PAK4), and the Notch regulator YAP1.^18^, ^19^, ^20^. The anti-tumor activity of anti-miR-221 and miR-199a-3p molecules on HCC has been demonstrated using *in vivo* mouse models.^16^, ^20^, ^21^, ^22^, ^23^

The availability of animal models that reproduce human liver carcinogenesis is of essential importance for preclinical testing. Many of such models have provided relevant information regarding molecular and pathological mechanisms of HCC.^24^, ^25^, ^26^ However, one limitation of the available animal models is that they commonly develop HCC in the absence of cirrhosis. This might negatively affect the assessment of novel systemic therapies with respect to their translation to typical human conditions. One well-established method to induce liver damage and promote fibrosis and cirrhosis in rats is a chronic exposure to the hepatotoxin carbon tetrachloride (CCl_4_).^27^ In mice, achieving advanced cirrhosis with ascites is more difficult, as prolonged exposure to the toxin affects animal survival.^28^ Domenicali and colleagues^29^ described the use of short-term inhalation cycles of CCl_4_ as an efficient method to induce decompensated cirrhosis in mice.

In the present study, we used the method of Domenicali et al.^29^ on TG221 transgenic mice to test the possibility of inducing liver tumors in the context of cirrhosis. The model was then used for investigating the prophylactic activity of anti-miR-221 as well as miR-199a-3p molecules.

## Results

### CCl_4_ Treatment Induces Cirrhosis and Ascitic Decompensation in Mice

**Figure 1:**
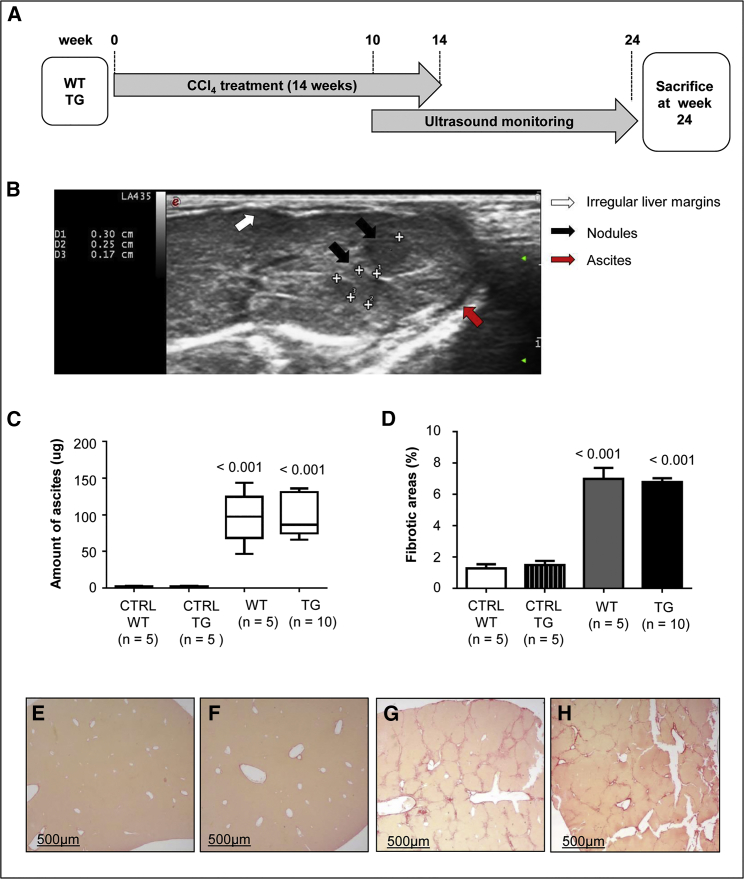
Carbon Tetrachloride Treatment Induces Cirrhosis in Wild-Type and TG221 Mice (A) Liver cirrhosis was induced in TG221 (TG) and wild-type (WT) mice by short carbon tetrachloride (CCl_4_) inhalation cycles, as described in the Materials and Methods. (B) Both WT and TG221 mice were monitored for the presence of hepatic lesions using an ultrasound diagnostic device during the weeks following the induction protocol. The presence of liver fibrosis and cirrhosis was evident in mice from both groups. The image is from a CCl_4_-treated TG221 mouse; ascites in the peritoneal cavity (red arrow) and irregular liver margins (white arrow) are shown. In addition, the presence of liver nodules was clearly evident (black arrows). (C) A significant accumulation in ascitic fluids in the peritoneal cavity, based on the total amount of ascites at the time of sacrifice, occurred in both WT and TG221 mice, with respect to that in the untreated control groups (p value < 0.001 for both groups). (D) Similarly, the quantification of fibrotic areas showed a comparable amount of liver fibrotic tissue between WT and TG221 mice, but a significant difference between these groups and control (CTRL) mice (p value < 0.001 for both groups). Data are represented as mean values ± SD. Representative images of Sirius Red staining, specific for collagen accumulation, in histological liver sections of untreated WT (E), untreated TG221 (F), CCl_4_-treated WT (G), and CCl_4_-treated TG221 (H) mice are shown. Scale bar, 500 μm. In the image, TG221 is indicated as TG.

TG221 (TG) and wild-type (WT) mice of the same background strain B6D2 (a cross between C57BL/6J [B6] × DBA/2J [D2]) were treated with CCl_4_, following an administration protocol described by Domenicali et al.,^29^ which consists of multiple short cycles of CCl_4_ inhalation (Figure 1A). During the 14-week induction phase, mice displayed no signs of suffering, as shown by an increase in body weight over time for both experimental groups (Figure S1).

WT and TG221 mice were monitored for the presence of hepatic lesions using an ultrasound device. Both groups of mice exhibited signs of liver fibrosis and cirrhosis, as shown by irregular liver margins and peritoneal effusions after 12–14 weeks of treatment (Figure 1B). All mice were sacrificed 10 weeks after the end of CCl_4_ treatment. Consistent with ultrasound imaging, the livers of all animals exposed to CCl_4_ had cirrhotic features with micronodular pale surfaces. Quantification of ascites revealed no significant differences between TG221 and WT animals, demonstrating the successful induction of cirrhosis in both groups. Upon histologic examination, Sirius Red staining showed bridging fibrosis in both TG221 and WT mice, but not in untreated mice (Figures 1C–1H). Moreover, activation of hepatic stellate cells was confirmed by the overexpression of α-smooth muscle actin (*α-Sma*), connective tissue growth factor (*Ctgf*), and transforming growth factor beta 1 (*Tgf-b1*) (Figure S2).

### CCl_4_ Treatment Leads to Cancer Formation in TG221 Mice

**Figure 2:**
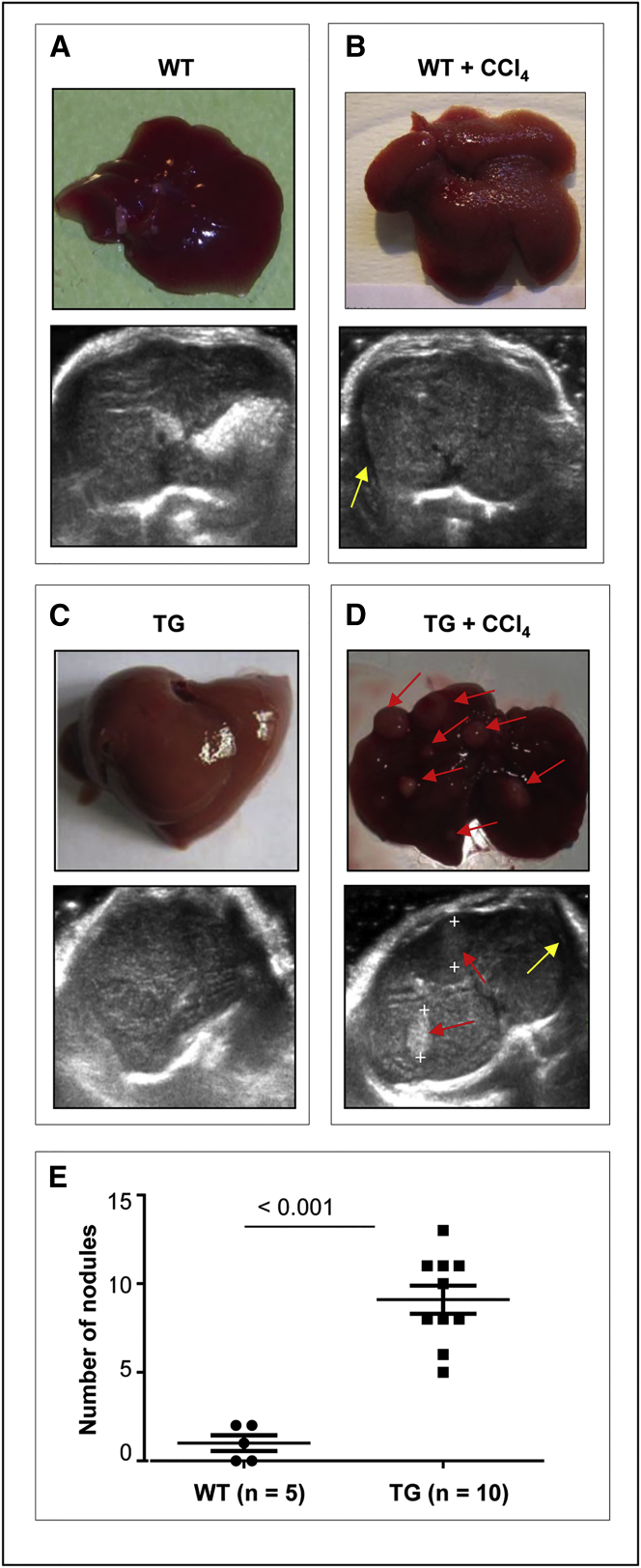
CCl_4_ Treatment Promotes Cirrhosis and Tumor Formation in the Livers of TG221 Mice Representative macroscopic and ultrasound images of livers from untreated WT (A), CCl_4_-treated WT (B), untreated TG221 (C), and CCl_4_-treated TG221 (D) mice. Livers of untreated TG221 and WT mice look a little different, as the aberrant expression of miR-221 in the liver of TG221 is responsible for an increase in volume and a pale exterior associated with steatohepatitis changes, as previously described.^16^ Livers of CCl_4_-treated mice presented a cirrhotic appearance with a granulated surface. At the time of sacrifice, macroscopic nodules were evident in the livers of 100% of TG221 mice (as indicated by red arrows), but not in WT mice. Ultrasound images show the presence of ascites (yellow arrows) in both CCl4-treated WT and TG221 mice, while liver nodules were only visible in TG221 mice (delimited by plus symbol and indicated by red arrows). Conversely, untreated WT and TG221 mice show homogeneous liver parenchyma, with no ascites or nodules. (E) The numbers of nodules detected by histopathological examination were significantly higher in TG221 mice than in CCl_4_-treated WT mice (p value < 0.001). In the image, TG221 is indicated as TG.

A significant difference in liver pathology was observed between WT and TG221 mice. At 4 weeks from the end of CCl_4_ treatment, ultrasound analysis revealed the presence of liver nodules in TG221, but not in WT, mice. Upon sacrifice, macroscopic nodules were indeed visible in the livers of all TG221 mice, but not in WT mice (Figures 2A–2D).

**Figure 3:**
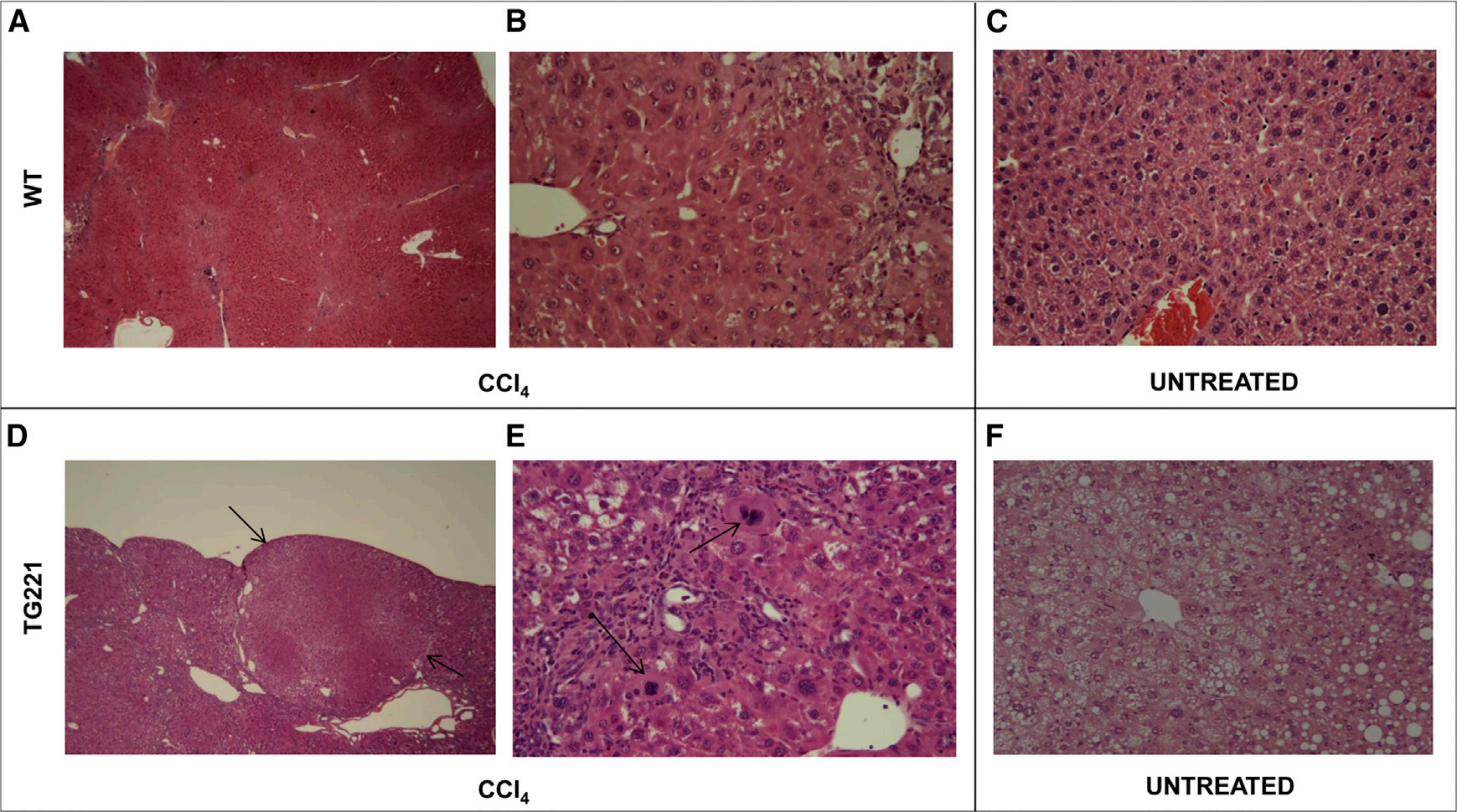
CCl_4_-Treated TG221 Mice Present Neoplastic Proliferative Lesions H&E-stained livers from untreated and CCl_4_-treated mice (200× and 40× magnification). In WT mice (A–C), alterations in the lobular structure of the liver and hepatic degeneration, with the presence of regenerative nodules typical of hepatic cirrhosis, were detected in CCl_4_-treated animals (A and B), but not in untreated mice (C). In TG221 mice (D–F), the same structural alterations in WT animals were observed in CCl_4_-treated animals (D and E), with a higher and more intense grade of inflammation. In addition, proliferative tumor lesions were present (black arrows, D) and associated with dysplastic aspects and atypical mitosis (black arrows, E), resembling hepatocellular carcinomas. (F) These alterations were absent in livers from untreated TG221 mice, albeit steatohepatitis changes, typical of the TG221 mouse,^16^ were observed.

Histological analyses confirmed a significant increase in the number and size of focal lesions in the livers of CCl_4_-treated TG221 mice compared to those in WT controls (Figure 2E). Most lesions in TG221 mice were dysplastic nodules or HCC, whereas in WT mice most were macro-regenerative or hyperplastic nodules (Table 1; Figures S3 and S4). The livers of WT CCl_4_-treated mice showed the loss of an ordered lobular architecture with bridging fibrosis. In this background, hepatocytes aggregated into regenerative-proliferative lesions, displayed typical mitosis, and did not contain dysplastic components (Figures 3A–3C). In TG221 mice, necro-inflammatory changes appeared to be more intense and extended, and the degenerative components of hepatocytes were marked and associated with both lithic and coagulative necrotic effects. Notably, progression to malignant tumors was detected based on the development of nodule-in-nodule proliferation, characterized by nuclear atypia, mitoses, increased trabecular width, and infiltrative growth (Figures 3D–3F; Figure S4).

**Table 1:** Histopathological Examination of Liver Tissues

Genotype	Mouse ID	Diagnosis
Wild-type	WT-1	hyperplasia
	WT-2	hyperplasia
	WT-3	hyperplasia
	WT-4	hyperplasia
	WT-5	hyperplasia
TG221	TG-1	dysplasia
	TG-2	HCC
	TG-3	HCC
	TG-4	dysplasia
	TG-5	HCC
	TG-6	HCC
	TG-7	dysplasia
	TG-8	HCC
	TG-9	HCC
	TG-10	HCC

**Figure 4:**
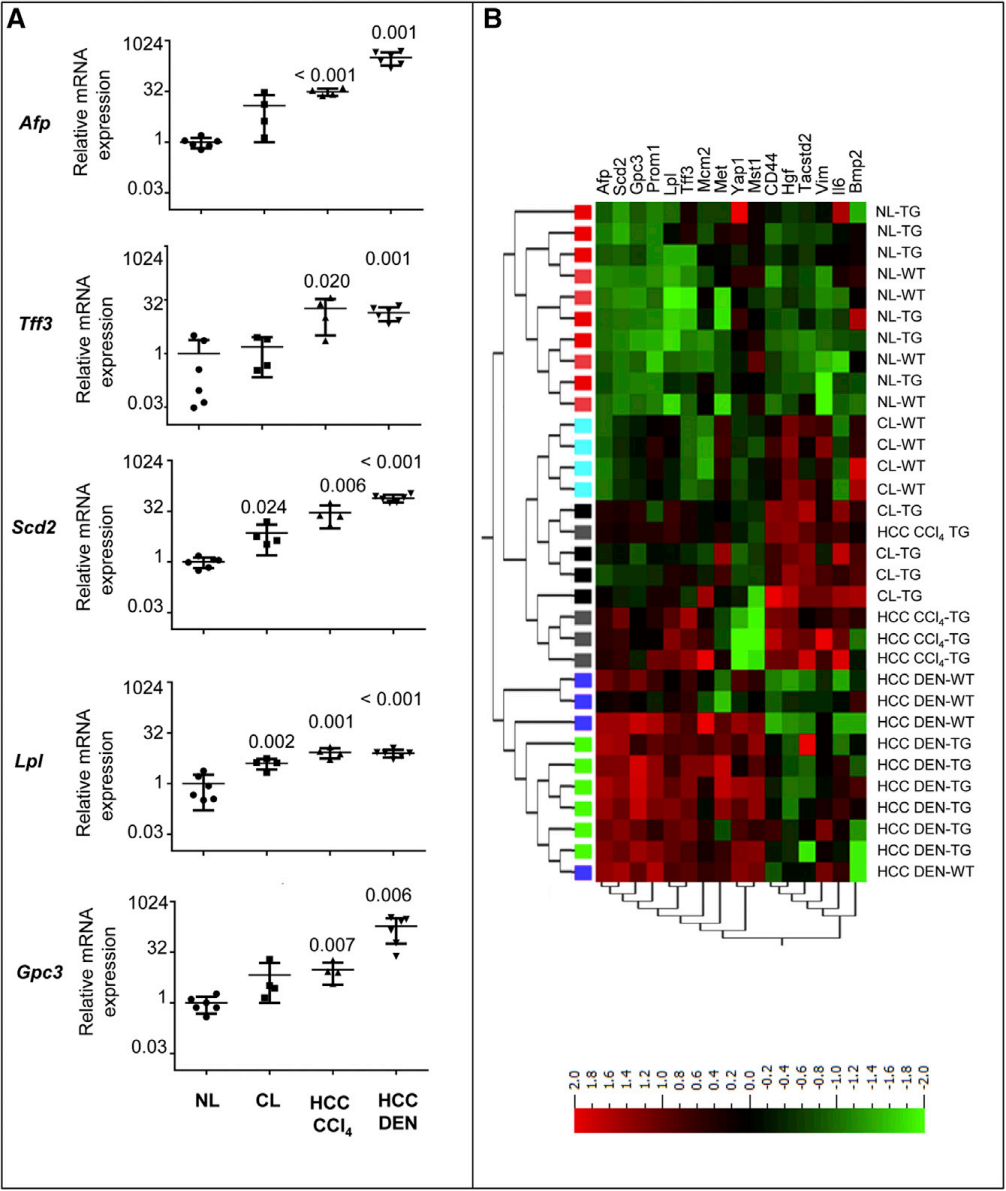
Tumor-Associated Genes Are Similarly Deregulated in CCl_4_-Induced and Diethylnitrosamine-Induced TG221 Liver Tumors (A) The tumor-associated genes *Afp*, *Tff3*, *Scd2*, *Lpl*, and *Gpc3* were highly expressed in TG221 liver tumors in both cirrhotic and non-cirrhotic conditions. A clear progression in their expression level was also observed from normal livers to cirrhotic livers and CCl_4_-induced or diethylnitrosamine (DEN)-induced HCC. The p values refer to mRNA expression levels in cirrhotic (CL) and HCC conditions versus those in normal livers (NL). The scale on the ordinate axis refers to mRNA expression related to that in normal livers. (B) Unsupervised hierarchical cluster analysis of gene expression to assess similarities between tumors arising in TG221 mice after DEN treatment versus those induced by CCl_4_ treatment. Most TG221 DEN-derived tumors (HCC DEN-TG) were found to be carcinomas. Normal livers (NL-WT), cirrhotic livers (CL-WT), and DEN-derived tumors (HCC DEN-WT) from WT mice were also included in this analysis. The analysis was performed using a list of genes described as markers of hepatic tumor progression as well as markers of proliferating and self-renewing liver cells. HCC CCl_4_ samples exhibited an expression profile that was highly similar to that of HCC DEN samples, whereas cirrhotic livers from TG221 mice (CL-TG) displayed an intermediate expression pattern between that of HCC and normal liver. Color legend is as follows: NL-WT, pink boxes; CL-WT, light blue boxes; HCC DEN-WT, blue boxes; NL-TG, red boxes; CL-TG, black boxes; HCC DEN-TG, green boxes; and HCC CCl_4_-TG, gray boxes. Abbreviations are as follows: *Afp*, alpha fetoprotein; *Scd2*, stearoyl-Coenzyme A desaturase 2; *Gpc3*, glypican 3; *Prom1*, prominin 1; *Lpl*, lipoprotein lipase; *Tff3*, trefoil factor 3; *Mcm2*, minichromosome maintenance-deficient 2 mitotin; *Met*, met proto-oncogene; *Yap1*, yes-associated protein 1; *Mst1*, macrophage-stimulating 1 (hepatocyte growth factor-like); *CD44*, CD44 antigen; *Hgf*, hepatocyte growth factor; *Tacstd2*, tumor-associated calcium signal transducer 2; *Vim*, Vimentin; *Il6*, interleukin-6; and *Bmp2*, bone morphogenetic protein 2. In the image, TG221 is indicated as TG.

In support of the malignant nature of tumors from CCl_4_-treated mice, we investigated the expression of genes, such as alpha-fetoprotein (*Afp*), trefoil factor 3 (*Tff3*), stearoyl-coenzyme A desaturase 2 (*Scd2*), lipoprotein lipase (*Lpl*), and glypican 3 (*Gpc3*), which are known markers of hepatic tumor progression.^30^, ^31^ As positive controls for mouse HCC, we used samples from control DEN-induced HCC obtained in TG221 mice in previous studies.^16^, ^20^ The level of expression was progressively increased from normal liver, cirrhotic tissue, CCl_4_-induced tumors, and DEN-induced HCC (Figure 4A).

In addition, by using a wider gene panel that included the above genes as well as additional genes described as markers of proliferating and self-renewing liver cells,^32^ we observed that, while the heatmap from an unsupervised analysis shows that the HCCs from CCl4-treated mice cluster with cirrhotic livers (Figure 4B; Table S1), it should also be noted that a group of genes (*Afp*, *Scd2*, *Gpc3*, *Prom1*, *Lpl*, *Ttf3*, and *Mcm2*) exhibit an expression profile more similar to DEN-induced HCC than to cirrhotic livers, thus indicating a progression to cancer of CCl_4_-induced tumors. These data indicate that these latter tumors maintain a cirrhotic signature but already evolved toward a malignant signature, thus confirming the results from histopathological analyses.

### Prophylactic Treatment with Anti-miR-221 Oligonucleotides or miR-199a-3p Mimics Prevents the Development of Malignant Lesions

**Figure 5:**
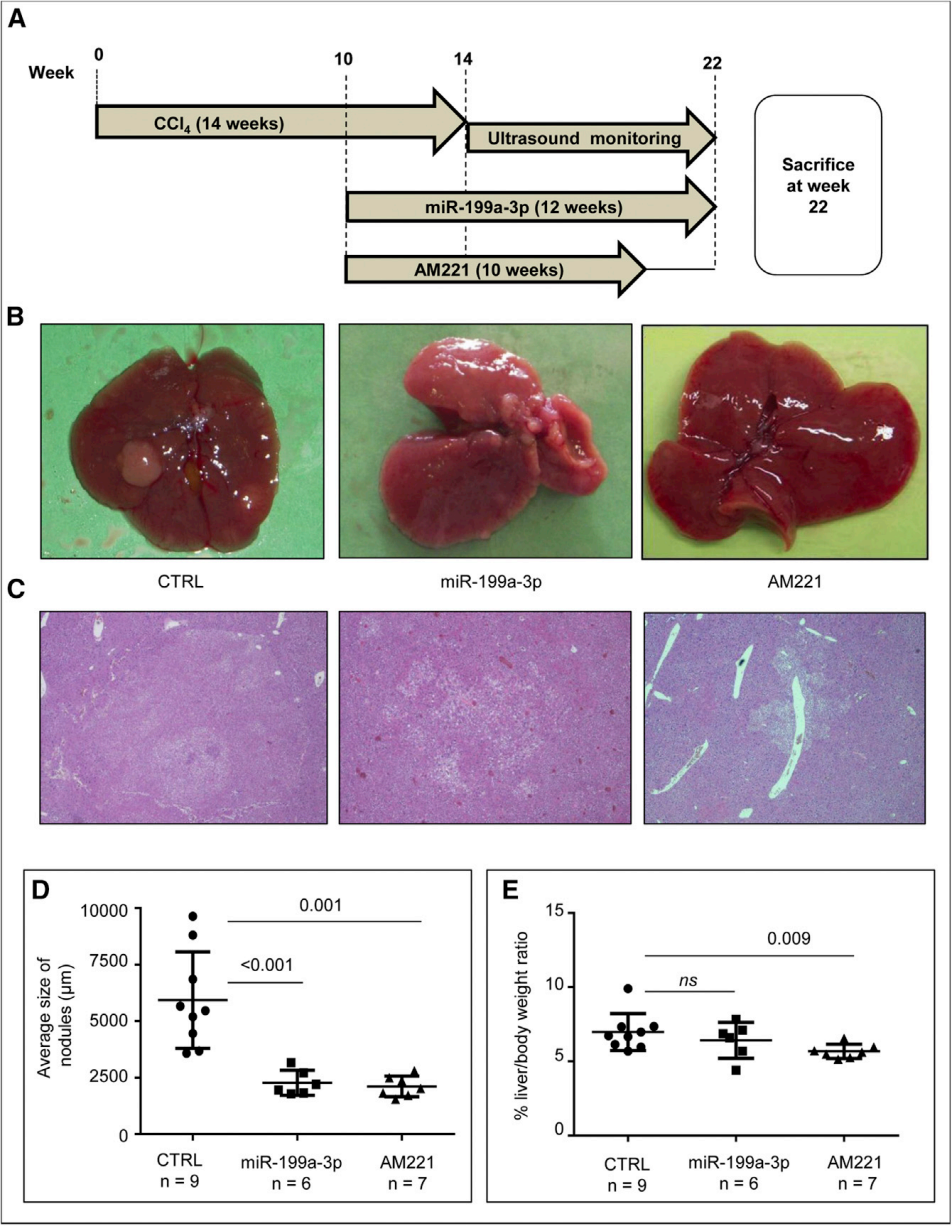
miR-199a-3p Mimics and Anti-miR-221 Molecules Exhibit Prophylactic Activity in the TG221 Model (A) The experimental design was subdivided into a CCl_4_ induction period of 14 weeks and a prophylactic miRNA-based treatment period. Specifically, after 10 weeks of CCl_4_ induction, one group of mice received a weekly dose of anti-miR-221 (AM221) for 10 weeks and another group received a dose of miR-199a-3p mimic three times per week for 12 weeks. AM221-treated mice were sacrificed 2 weeks after the last treatment, due to the longer stability of the anti-miRNA molecules, whereas miR-199a-3p-treated mice were sacrificed 24 hr after the last mimic injection. A group of mice treated with a scramble oligonucleotide was employed as the negative control (CTRL). (B) Representative macroscopic images of livers from scramble-treated mice (CTRL), miR-199a-3p-treatd mice (miR-199a-3p) and AM221-treated mice (AM221) at the time of sacrifice. (C) Hematoxylin and eosin stained livers from the same mice described above (40× magnification). Liver nodules in control animals showed malignant progression to hepatocellular carcinoma (HCC) accompanied by a loss of fatty changes, increased nuclear: cytoplasmic ratio, nuclear atypia, and easily detectable mitotic activity; treated mice had dysplastic nodules with fatty and focal nodular hyperplasia-like changes. (D) The panel shows the average size of tumor lesions in each mouse and demonstrates how they are significantly reduced after miRNA-therapy treatment. (E) Liver-to-body weight ratios are lower (p value = 0.009 in AM221-treated animals) or tended to be lower (p value not significant in miR-199a-3p-treated mice) in treated animals than in controls.

As the model used herein recapitulates the phases of human HCC arising in cirrhotic liver, we investigated the ability of miRNA-based molecules to prevent or control cancer development. Three experimental groups were designed: (1) mice treated with miR-199a-3p, (2) mice treated with anti-miR-221 (AM221), and (3) a control group treated with a scrambled oligonucleotide. Treatments were performed according to a protocol shown in Figure 5A. During treatments, mice were monitored by ultrasound to evaluate tumor appearance and growth. Compared to controls, we observed a reduction in tumor growth, including the complete inhibition or regression of tumor nodules, in the group of animals treated with miRNA-based molecules (Figure S5).

At the time of sacrifice, tumor nodules in the livers of mice treated with miRNA-based molecules were macroscopically smaller than those in the control group (Figures 5B–5D). A reduction in the liver-to-body weight ratio observed in animals treated with AM221 or miR-199a-3p, compared to that in controls, confirmed the reduced tumor burden (Figure 5E).

Histological analyses confirmed that the livers of miRNA-treated animals contained a smaller number of proliferative foci. Notably, HCCs were detected in at least four of the nine control mice, whereas only one HCC was detected among all miRNA-treated mice (Table S2). Treated mice also showed evidence of reduced liver damage, as assessed by hydropic changes, number of necro-inflammatory foci, and/or confluent necrosis (Figure 5C). A reduction in the level of *Gpc3* expression could also be noted in livers of AM221-treated mice (Figure S6).

**Figure 6:**
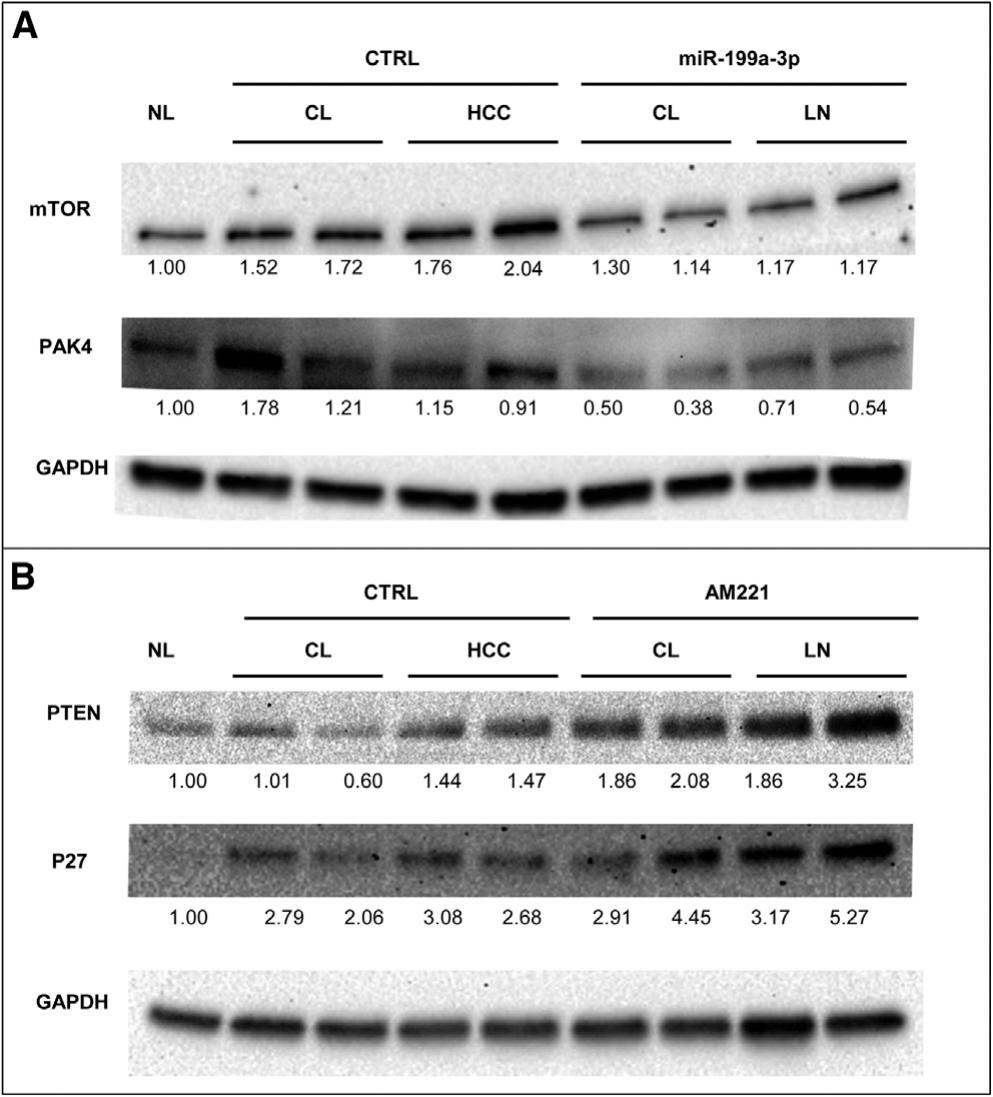
miR-199a-3p and miR-221 Targets Are Deregulated in miRNA-Treated TG221 Mice Western blot analysis was performed on cirrhotic tissues (CL) and liver nodules (LN) extracted from mice treated with anti-miR-221 (AM221) and miR-199a-3p mimic molecules. The *in vivo* activity of these molecules was confirmed by the deregulation of important target proteins, as compared to expression in the scramble-treated control group (CTRL). We observed a reduction in the mechanistic target of rapamycin (mTOR) and p21-activated kinase 4 (PAK4) in tumors from miR-199a-3p-treated mice (A), and an increase in phosphatase and tensin homolog (PTEN) and cyclin-dependent kinase inhibitor 1B (P27KIP1) in tumors from AM221-treated mice (B). Protein expression data were normalized versus levels of glyceraldehyde-3-phosphate dehydrogenase (GAPDH), and fold-change values with respect to normal livers (NL) were reported.

In parallel with the observed macroscopic and microscopic changes, miR-199a-3p and anti-miR-221 were found to induce molecular changes. Indeed, the levels of protein targets of these miRNAs were changed, as assessed by the western blot analysis of cirrhotic tissues and tumor nodules. Specifically, the downregulation of mTOR and PAK4 was observed after treatment with miR-199a-3p (Figure 6A); the upregulation of PTEN and cyclin-dependent kinase inhibitor 1B (CDKN1B -P27) was also observed after treatment with AM221 (Figure 6B). These results confirmed that miRNA-based treatments were associated with molecular effects on important cancer-associated pathways.

## Discussion

This work describes a new mouse model that recapitulates the progress of chronic liver disease from fibrosis and cirrhosis to HCC and miRNA-based approaches for the prevention of liver cancer in a cirrhotic liver. This is particularly relevant as approximately 80% of HCCs occur in patients with an underlying cirrhosis.

The availability of an optimal animal model that faithfully mimics the human disease can help to achieve results applicable to humans. Current HCC mouse models do not generally encompass liver fibrosis and cirrhosis. Treatment with CCl_4_ was previously shown to induce liver fibrosis and cirrhosis in rats and mice, but only sporadically did animals develop liver cancer. Experimental models of decompensated cirrhosis are well established in rats,^28^, ^33^, ^34^ but only additional repeated injections of low-dose DEN result in the development of HCC in these animals.^35^, ^36^ Mouse models that develop HCC in a background of fibrosis or cirrhosis have also been produced by a combination of DEN and CCl_4_ induction.^37^, ^38^, ^39^ However, prolonged exposure to the hepatotoxin negatively affects animal survival.^28^ Additional models of HCC concomitant with liver cirrhosis included CCl_4_ administration combined with orthotopic tumor implantation or with adenoviral Cre-recombinase injection in genetically engineered mouse models.^40^ In a more recent study, the concomitant CCl_4_ treatment with the injection of transposons expressing *Myc* and a short hairpin RNA that downregulates p53 (shp53) resulted in 100% HCC incidence accompanied by liver fibrosis.^41^

The TG221-CCl_4_ model reported here is based on a simpler approach, as it only requires CCl_4_ administration, using the method described by Domenicali et al.,^29^ to induce decompensated cirrhosis in the TG221 mouse strain. TG221 mice develop cirrhosis with ascitic decompensation, accompanied by cancer development in 100% of animals. Liver histology revealed disrupted lobular architecture, bridging fibrosis, and necro-inflammatory liver damage. Liver fibrogenesis was confirmed by the increased deposition of collagen and the increased expression of *α-Sma*, a specific marker of hepatic stellate cell activation,^42^ in addition to the upregulation of *Tgf-β* and *Ctgf* pro-fibrotic molecules.^43^, ^44^ The neoplastic nature of the hepatocellular nodules was confirmed by histology, which identified a spectrum of lesions ranging from dysplastic foci or nodules to HCC. The expression of genes such as *Afp*, *Tff3*, *Lpl*, *Scd2*, and *Gpc3*, which are involved in liver carcinogenesis, further supported the presence of a malignant phenotype.

Because the TG221-CCl_4_ mouse recapitulates all the phases of chronic liver disease, from fibrosis to HCC, it provides a new excellent model for testing treatments aimed at the prevention of HCC. Chemopreventive agents may act at various phases of chronic liver disease to prevent its progression to HCC. In humans, immunization against hepatitis B virus (HBV) and antiviral therapy against HBV and hepatitis C virus (HCV) have been associated with reduced HCC risk. In addition, statins, aspirin, and the anti-diabetic agent metformin have also shown promising chemopreventive activity.^45^, ^46^ In animals, most of the studies were carried out in the therapeutic setting;^47^ nonetheless, a number of compounds exhibited chemopreventive effects. They included natural plant products such as curcumin, resveratrol, epigallocatechin, caffeine,^48^, ^49^, ^50^ anti-fibrotic agents,^51^ COX-2 inhibitors,^52^ and S-adenosylmethionine.^53^

Here we tested miRNA-based molecules for the prevention of liver cancer. The restoration of tumor suppressor miRNAs or inhibition of oncogenic miRNAs have been previously tested in the therapeutic experimental setting in a number of pre-clinical models, including liver cancer.^54^, ^55^ Based on previous studies, miR-221 and miR-199a-3p represented suitable candidates.^16^, ^20^ The importance of miR-221 to liver tumorigenesis was described in orthotopic and transgenic HCC mouse models,^16^, ^56^ and its oncogenic function was associated with the ability to promote cell proliferation and inhibit apoptosis.^13^, ^14^ As such, silencing miR-221 *in vivo* was found to reduce tumor growth, increase mouse survival,^16^, ^57^ and inhibit the establishment of hepatoma xenografts and lung metastasis in nude mice.^58^ miR-199a-3p is one of the most highly expressed miRNAs in normal liver and is downregulated in virtually all HCCs.^17^, ^22^ This miRNA has a pivotal role in several cancer-associated pathways.^19^, ^22^, ^59^, ^60^ The restoration of miR-199a-3p expression in subcutaneous or orthotopic HCC mouse models demonstrated its anti-tumor activity, suggesting that miR-199a-3p replacement might represent a promising therapeutic strategy to treat HCC.^20^, ^22^, ^59^, ^60^, ^61^, ^62^ In this work, we focused on the ability of each of these miRNAs to prevent tumor development using the TG221-CCl_4_ mouse model. Systemic administration of either anti-miR-221 or miR-199a-3p mimics resulted in a reduction in the malignant progression of hepatocellular nodules.

Biological outcomes were accompanied by detectable molecular effects that allowed us to recognize and confirm cancer-associated signaling pathways regulated by miR-199a-3p and miR-221.^14^, ^15^, ^18^, ^22^ In fact, the enforced expression of miR-199a-3p elicited the downregulation of mTOR and PAK4 proteins, while the suppression of miR-221 by anti-miRNA caused the upregulation of PTEN and CDKN1B. PAK4, a serin-threonine kinase member of the PAK family,^63^ is at the crossroads of several oncogenic pathways. It functions as Rho’s GTPase effector to reorganize the cytoskeleton: it is indeed activated by Cdc42 to promote cell motility through the formation of lamellipodia and filopodia. PAK4 can also act on other targets: it opposes the activation of caspase 8, promotes stabilization and activation of β-catenin, and through its scaffold function stimulates AKT activation.^64^, ^65^, ^66^, ^67^ In a variety of human cancers, PAK4 is overexpressed, amplified, or is affected by mutations that activate its kinase activity.^68^, ^69^, ^70^, ^71^, ^72^ mTOR, an essential protein for the activation of the PI3K-AKT pathway, is a serin-threonine kinase that, in complex with RICTOR, phosphorylates and activates AKT; in complex with RAPTOR, it is phosphorylated by AKT to regulate protein synthesis, growth, proliferation, and cell survival.^73^

The PI3K-AKT pathway is frequently activated by mutations of genes such as RAS, PI3KCA, or PTEN, which are, however, rare in HCC. In HCC, in addition to miRNA deregulation, this pathway is instead activated by numerous growth factors, such as hepatocyte growth factor (HGF), platelet-derived growth factor (PDGF), and vascular endothelial growth factor (VEGF), present in the tumor microenvironment, or by the amplification of genes coding for fibroblast growth factor (FGF; in particular FGF19, 5%–14% of HCC cases).^74^ PTEN, also a key protein in the regulation of the PI3K-AKT pathway, is a phosphatase that blocks AKT activation by dephosphorylating the phosphatidylinositol (3,4,5)-trisphosphate (PIP3) to PIP2. Inactivating mutations and deletions of the *PTEN* gene are frequent in human tumors.^75^ In preclinical models, the importance of PTEN in the development of HCC was demonstrated by a mouse model in which the *Pten* gene was inactivated, causing steatohepatitis at 10 months of age and HCC in 100% of animals at 18 months.^76^ The *CDKN1B* gene encodes the cyclin-dependent kinase p27 inhibitor. It controls the progression of the cell cycle G1 phase by binding to cyclinD-CDK4 and cyclinE-CDK2. It is, therefore, considered a tumor suppressor, and it is dysfunctional in cancer through various mechanisms.^77^

Overall, our results suggest that the action of miR-199a-3p and anti-miR-221 in preventing the emergence of HCC occurs through the control of multiple cancer-associated molecular pathways, which include PI3K-AKT, WNT-β-catenin, cell cycle, invasion, and motility. There are no apparent effects on fibrosis and cirrhosis processes, as suggested by the stable expression of α-Sma after miRNA challenges. Findings from this study provide the basis for the use of miRNA-based therapeutics to prevent liver cancer, especially considering that no apparent toxic effects were detected in treated mice.

### Conclusions

This study describes the development of a mouse model, based on the TG221 strain, which represents an accurate preclinical example of hepatocarcinogenesis in a background of cirrhosis, a condition that mirrors the pathogenesis of most human HCCs. This model could be used to investigate the mechanisms of hepatocarcinogenesis in the cirrhotic liver and to develop preclinical approaches to prevent or treat liver cancer that arises in the context of cirrhosis.

Here, by testing anti-miRNA and miRNA mimics as prophylactic molecules, we demonstrated that miRNA-based treatments did not cause apparent toxicity, resulted in a reduction in tumor nodule size, and, most importantly, prevented the malignant transformation of nodular lesions. These results suggest that the tested molecules have the potential to reduce the risk of HCC in individuals with cirrhosis, the main risk factor for HCC in humans.

## Materials and Methods

### *In Vivo* Studies

The transgenic mouse TG221 has been previously described.^16^ Both TG221 and WT mice have the same strain B6D2 (a cross between C57BL/6J [B6] × DBA/2J [D2]) background. Mice were maintained in vented cabinets at 24°C with a 12-hr light-dark cycle and with food and water *ad libitum*. All animals were sacrificed under inhalational anesthetic using isoflurane to minimize suffering. Mice were subjected to necropsy and tissues were partly fixed in 10% formalin and partly frozen in liquid nitrogen. This study was carried out in strict accordance with the Guidelines for the Care and Use of Laboratory Animals, and the experimental protocols were approved by the Italian Ministry of Health. To comply with the 2010/63/EU directive of the European Parliament and Council, enforced by the Italian law requiring a minimized number of experimental animals, G*Power (http://www.gpower.hhu.de/) was used to determine the sample size. All animals were randomly assigned to different treatment groups at the start of the study. Frozen liver HCCs of DEN-treated control TG221 mice were obtained from our laboratory tissue bank. Mice were treated as previously described.^20^

### Induction of Cirrhosis

Liver cirrhosis was induced in both TG221 and WT male mice by short CCl_4_ (Sigma-Aldrich, St. Louis, MO, USA) inhalation cycles, as previously described,^29^ with some modifications (because of differences in animal strains). Briefly, WT (5 mice) and TG221 animals (10 mice) were subjected to short-term inhalation of CCl_4_ via a flowmeter three times weekly for 14 weeks. A group of WT (5 mice) and TG221 (5 mice) animals was used as an untreated control. TG221 mice were subjected to a reduced induction protocol (1 L/min), because they did not tolerate standard CCl_4_ treatment (2 L/min). The treatment started at 5–6 weeks of age. Phenobarbital (0.3 g/L) was also administered in the drinking water to enhance CCl_4_ hepatotoxicity. After the 14-week standard induction, mice were monitored for the presence of hepatic lesions using an ultrasound diagnostic device (Philips IU22). For ultrasound analysis, mice were sedated via the intramuscular administration of a ketamine (100 mg/kg) and xylazine (10 mg/kg) solution. All mice with lesions, as documented by ultrasound, were sacrificed, and liver tissue samples were collected at weeks 22–24 for histological and molecular analyses. Tumor volume measurement was calculated according to the following formula: V = (4/3) × π × (L/2) × (L/2) × (D/2), where L = length and D = depth of the tumor. Data were obtained by analyzing section images from ultrasonographic examination videos.^78^

### Quantification of Ascites

At the time of sacrifice, a laparotomy was performed, and four strips of absorbing paper (5 × 30 mm) were placed in the abdominal cavity and removed after 3 min. The amount of ascites was calculated as the difference in the strip weight before and after placement in the abdominal cavity.

### miRNA-Based Treatments

To evaluate miRNA-based approaches to prevent HCC in the TG221 mouse model, an anti-miRNA oligonucleotide targeting miR-221 (Integrated DNA Technologies, Coralville, IA, USA) and an oligonucleotide that mimics the miR-199a-3p (Axolabs, Kulmbach, Germany) sequence were used. Specifically, the miRNA sequences were as follows: (1) anti-miR221, 5′-mG*mA*mAmAmCmCmCmAmGmCmAmGmAmCmAmAmUmG mU*mA*mG* mC*mU-3′ (where “m” represents 2′O-methyl RNA bases and “*” represents a phosphothioate bond); and (2) miR-199a-3p, 5′-ACAGUAGUCUGCACAUUGGUUA-3′ (unmodified sequence). Based on the experimental design, all mice received 14 weeks of CCl_4_ treatment. From the tenth week of treatment, mice were subdivided into three groups as follows: seven mice received a weekly dose of anti-miR-221 (5 mg/kg) for a period of 10 weeks, six mice received a dose of miR-199a-3p mimic (5 mg/kg) three times per week for a period of 12 weeks, and nine mice were treated with a scramble oligonucleotide. *In vivo* delivery was performed systemically (via intraperitoneal [i.p.] injection) using lipid nanoparticles as the vehicle.

### Lipid Nanoparticles

The lipid components of the nanoparticles were 1,2-dioleoyl-sn-glycero-3-phosphoethanolamine (DOPE); 1,2-dimyristoyl-sn-glycerol, methoxypolyethylene glycol (DMG-PEG, molecular weight [MW] 2,000; Avanti Polar Lipids, Alabaster, AL, USA); and linoleic acid (Sigma-Aldrich). The molar ratio of DOPE:linoleic acid:DMG-PEG was 50:48:2. The preparation of empty nanoparticles was performed as previously described.^79^

### Histological Procedures

Tissue samples from at least two representative fragments of each liver lobe were taken at necropsy, fixed in 10% phosphate-buffered formalin for 12–24 hr, and then embedded in paraffin. Serial 4-μm sections were stained with H&E to histologically determine the number and size of nodules. For Sirius Red staining, 4-μm liver tissue sections were deparaffinized, rehydrated, and then stained for 1 hr in saturated picric acid with 0.1 Sirius Red F3BA (Aldrich Chemicals, St. Louis, MO, USA) at room temperature. Next, the slides were washed twice with acetic acid solution and finally dehydrated in a graded alcohol series. Sections were evaluated using an image cytometer consisting of a single 2/3-in charge-coupled device (CCD) color camera (JVC Professional Europe, London, UK) mounted on a Leica DMLB microscope (Leica Microsystems, Wetzlar, Germany) equipped with a motorized scanning table (Märzhäuser, Wetzlar, Germany) controlled by Cytometrica software (C&V, Bologna, Italy). The fibrotic area was quantified based on four different fields (acquired at low magnification, 2.5× ) for each slide using ImageJ Software (https://imagej.nih.gov) and expressed according to the following formula: [collagen area/(total area − vascular lumen area)] × 100.

### Morphological Criteria Used for the Classification of Liver Nodules

Discrete hepatocellular foci (with a diameter <500 μm) that were cytologically distinguished from the surrounding liver and with expansile, permeative growth toward the surrounding hepatocellular plates were classified as dysplastic foci. These were generally localized around central veins and characterized by enlarged hepatocytes with fatty changes and/or eosinophilic, dense cytoplasm, with deposits of hyaline substances or globules. Similar lesions with diameter r > 500 μm were classified as dysplastic nodules (Figure S3). The same criteria, although with a size cutoff of 1,000 μm, are used in human pathology.^80^ Malignant transformation was defined by the development of nodule-in-nodule proliferation, with nuclear atypia, an increased nuclear:cytoplasmic ratio, mitotic activity, and increased trabeculae width (Figure S4).^81^

### RNA Extraction and qPCR Analysis

The total RNA fraction was obtained from samples using Trizol Reagent (Invitrogen, Carlsbad, CA, USA). RNA quality was assessed using the Agilent 2100 Bioanalyzer (Agilent Technologies, Santa Clara, CA, USA). Quantification of mRNA expression was performed using EvaGreen-based droplet digital PCR (ddPCR). 200 ng total RNA was retro-transcribed using random hexamers and oligo dT. After performing an appropriate dilution, 1 μL cDNA was used for amplification in a 20-μL reaction volume containing ddPCR EvaGreen Supermix and specific primers (1864033; Bio-Rad, Hercules, CA, USA). Droplet generation, cycling conditions, and the enumeration of positive droplets were performed according to previously described procedures.^82^ To normalize the relative abundance of miRNAs, we used the *β-actin* gene. For primer sequences, see Table S3.

### Gene Expression

RNA was hybridized to an Agilent Whole Mouse Gene Expression Microarray (G4852A, 8 × 60K; Agilent Technologies), and one-color gene expression was performed according to the manufacturer’s protocol. Labeled cRNA was synthesized from 100 ng total RNA using the Low RNA Input Linear Amplification kit (Agilent Technologies) in the presence of cyanine 3-cytosine triphosphate (CTP; PerkinElmer, Boston, MA, USA). Images generated by the Agilent scanner and Feature Extraction 10.5 software (Agilent Technologies) were used to obtain the microarray raw data. Qlucore Omics Explorer software (QOE) (http://www.qlucore.com/; Qlucore, Lund, Sweden) was used to analyze the microarray data.

### Western Blot Analyses

Tissue samples were collected and immediately frozen in liquid nitrogen. Samples were homogenized using a syringe in radioimmunoprecipitation (RIPA) buffer (R0278; Sigma-Aldrich) containing phosphatase and protease inhibitors (P2850 and P8340; Sigma-Aldrich) and processed following the manufacturer’s instructions. Rabbit antibodies against mTOR (7C10, 2983; Cell Signaling Technology, Danvers, MA, USA), PAK4 (3242; Cell Signaling Technology), PTEN (9552; Cell Signaling Technology), and p27 Kip1 (2552; Cell Signaling Technology) were diluted in 5% w/v BSA (A4503, Sigma-Aldrich), 1× Tris-buffered saline (TBS), and 0.1% Tween20 (Bio-Rad) and incubated at 4°C for 16 hr. An anti-glyceraldehyde-3-phosphate dehydrogenase (GAPDH) monoclonal antibody (clone 2D9, TA802519; OriGene) was used as a housekeeper. For chemiluminescent detection, a horseradish peroxidase-conjugated secondary antibody (7074; Cell Signaling Technology) was used in combination with Clarity Western ECL Blotting Substrate (170-5060; Bio-Rad) for signal detection. Digital images were acquired using a Chemidoc (Bio-Rad), and signals were quantified with ImageJ software. Protein expression levels were normalized according to the expression of the housekeeping protein.

### Statistical Analysis

Differences between groups were analyzed using a 2-tailed Student’s t test. A p value threshold <0.05 was considered significant. When appropriate, group value was expressed in terms of mean ± SD. GraphPad Prism 6.0 (GraphPad, La Jolla, CA, USA) was used for all data analysis. No samples or animals were excluded from the analyses, and none of the investigators were blinded to group allocations.

## Author Contributions

E.C., M.D., S.S., and M.N. contributed to overall conception and study design. E.C., M.D., L.D., P.G., F.G., M.B., C.B., R.C.S., B.K.E., B.Z., M.F., and F.F. performed all the experiments and the acquisition, analysis, and interpretation of data. E.C. and M.N. wrote the manuscript, which was edited by all co-authors. G.A., S.B., E.M.S., and L.G. contributed to a critical revision of the manuscript.

## Conflicts of Interest

All the authors declare no conflicts of interest.

## Supplemental Information

Document S1. Figures S1–S6 and Tables S1–S3

Document S2. Article plus Supplemental Information
