# Supplementary material for: MicroRNA-Based Prophylaxis in a Mouse Model of Cirrhosis and Liver Cancer
Source: Mol Ther Nucleic Acids. 2018 Dec 6;14:239–50. doi: 10.1016/j.omtn.2018.11.018 (PMC6330511; doi:10.1016/j.omtn.2018.11.018)
Supplement: Document S1. Figures S1–S6 and Tables S1–S3 [file mmc1.pdf]

OMTN, Volume *14*

Supplemental Information

MicroRNA-Based Prophylaxis

in a Mouse Model of Cirrhosis and Liver Cancer

Elisa Callegari, Marco Domenicali, Ram Charan Shankaraiah, Lucilla D'Abundo, Paola Guerriero, Ferdinando Giannone, Maurizio Baldassarre, Cristian Bassi, Bahaeldin K. Elamin, Barbara Zagatti, Manuela Ferracin, Francesca Fornari, Giuseppe Altavilla, Stella Blandamura, Enrico Maria Silini, Laura Gramantieri, Silvia Sabbioni, and Massimo Negrini

SUPPLEMENTAL INFORMATIONS

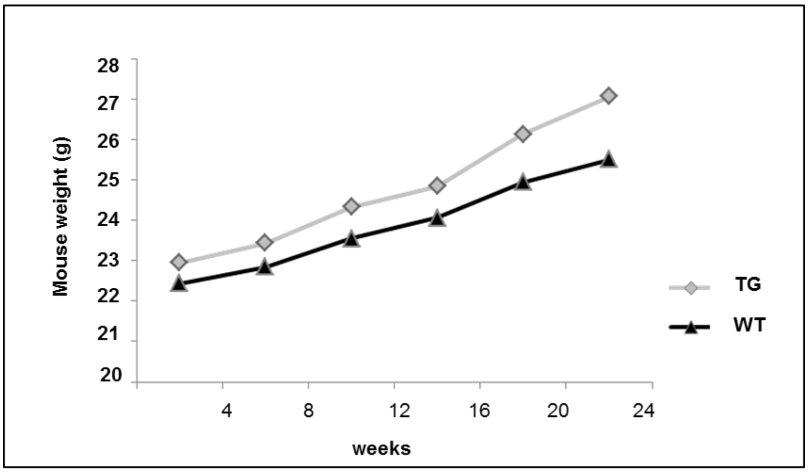

Figure S1. Weight increase in CCl_4-treated mice. During the CCl_4 induction phase, mice were monitored weekly and weighed to assess health status. An increase in body weight over time in both wild-type (WT) and transgenic TG221 (TG) animals was observed, indicating no distress or suffering due to treatment. Points in the graph represent the average weights of both experimental groups (in grams) every 4 weeks.

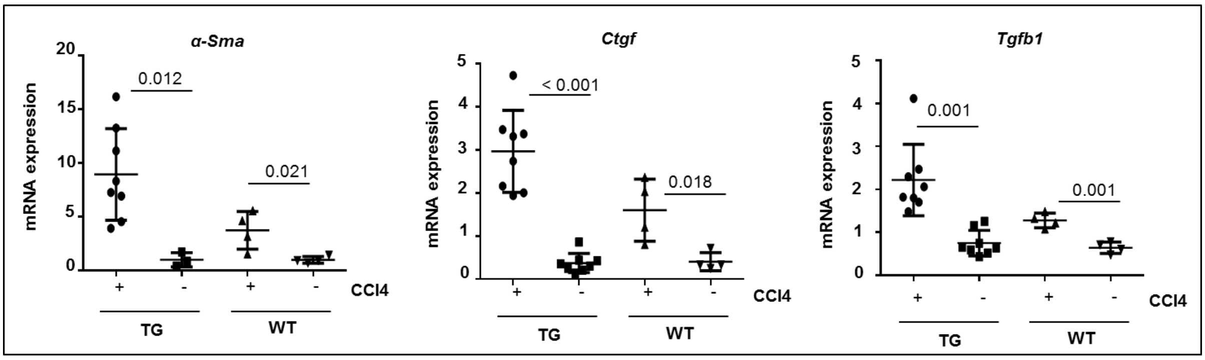

Figure S2. Increased levels of hepatic fibrosis markers are observed in CCl_4-treated TG221 mice. Quantitative PCR analysis of liver tissues from TG221 (TG) and wild type (WT) mice, showing an increase in the mRNA expression of alpha smooth muscle actin (*α-Sma*), connective tissue growth factor (*Ctgf*), and transforming growth factor beta (*Tgfb*1) in CCl_4-treated animals, as compared to that in control mice (p values are reported in the plots).

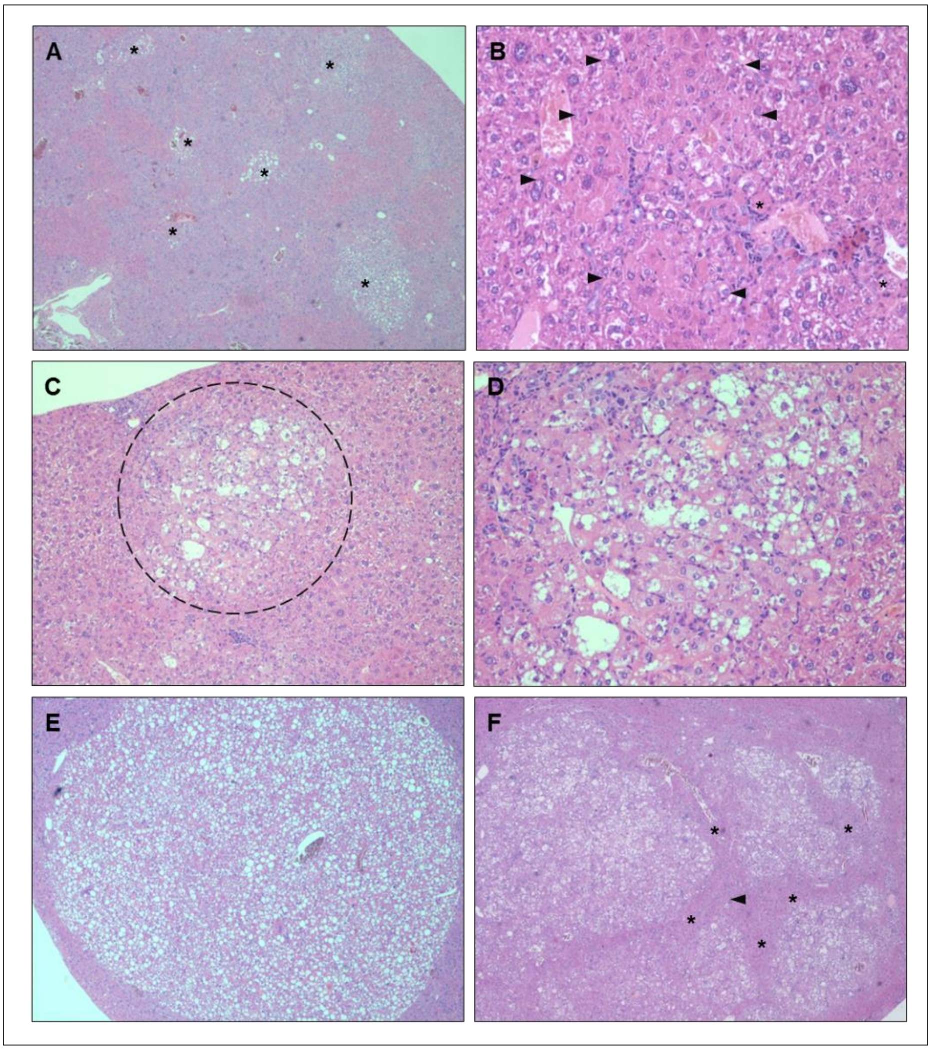

Figure S3. Spectrum of liver focal lesions observed in CCl_4-treated TG221 (TG) mice. Dysplastic foci and dysplastic nodules were arbitrarily distinguished based on size, using a 500 micron cut-off. The 500 micron threshold roughly corresponds to the eye-sight resolution for identifying a focal lesion. The same criteria, although with a size cut-off of 1000 micron, are used in human pathology. See *Morphological criteria used for the classification of liver nodules*, a section of Materials and Methods. (**A**) A variable number of minute, sub-acinar, steatotic foci (asterisks) centered around terminal veins were observed in CCl_4-treated TG mice (40×). (**B**) In the background liver, we observed hepatocytes with hydropic changes and necrosis in centrilobular areas, which were "spotty" and confluent (arrow points) (200×). (**C**) The steatotic foci progressively increased in size and formed discrete nodules with dysplastic features (selected area) (100×). (**D**, detail of panel C) In most nodules, a variable admixture of cells with fatty and 'balloon' changes and cells with eosinophilic dense cytoplasms with Mallry hyaline deposition and 'pale body' inclusions were observed (200×). (**E**) These nodules had no capsule, included peripheral portal triads, and showed replacement growth of the adjacent liver plates (40×). (**F**) Some nodules were separated by fibrous septa (asterisks) containing reactive bile ductules that arose from a central scar (arrow point); these features are similar to those observed in focal nodular hyperplasia (FNH) of the liver (40×).

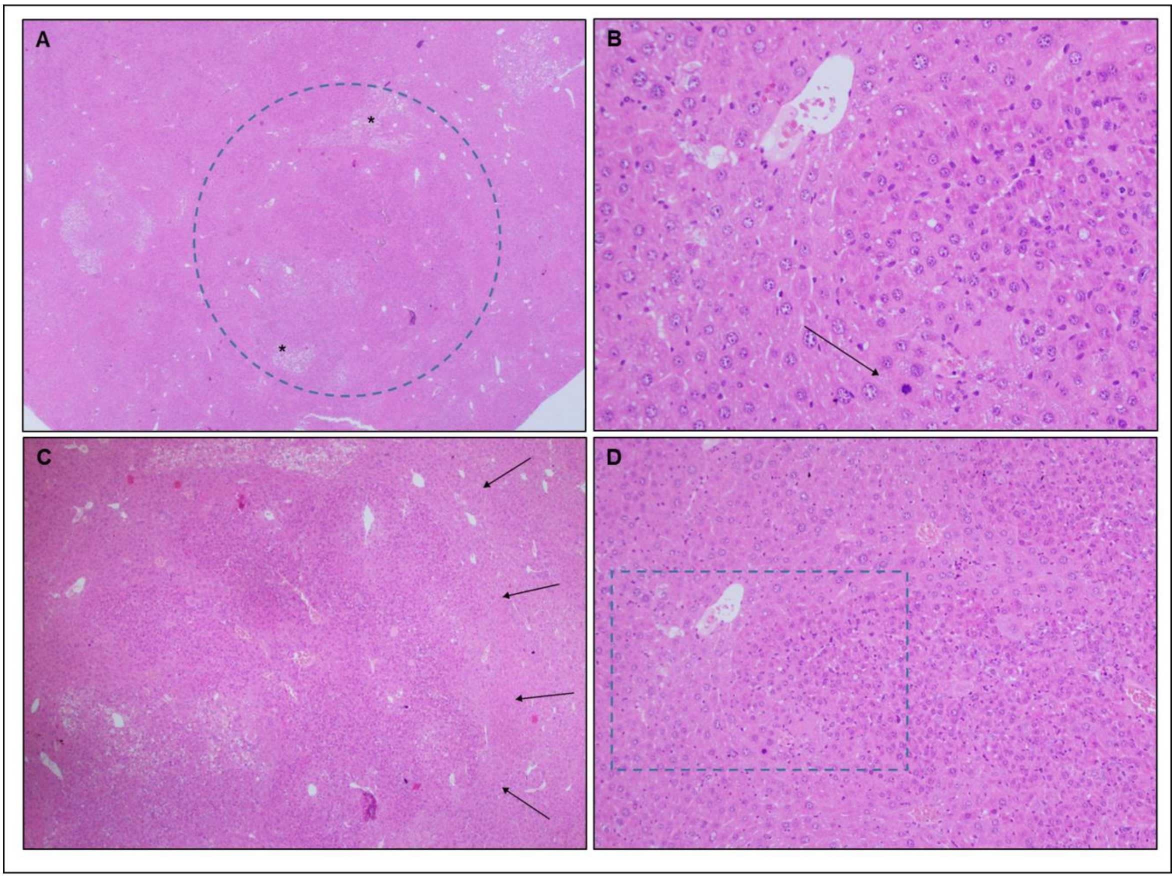

Figure S4. Progression of dysplastic nodules to early HCC. (**A**) A proliferative hepatocellular nodule (selected area), approximately 600 micron in maximum diameter, that developed in a setting of multiple steatotic foci. At periphery, two residual areas of adenomatous change with steatosis can be seen (asterisks). (**B**, detail of panel A) In the middle, cells lack the steatotic changes, show hyperchromatic nuclei with mitotic figures (arrow) and grow in laminae of increased width with loss of an orderly vascular pattern. (**C**) The cells permeate the surrounding hepatocyte laminae (arrows) and (**D**) progressively replace the dysplastic hepatocytes (selected area), as it occurs in early HCC development. See also *Morphological criteria used for the classification of liver nodules*, a section of Materials and Methods.

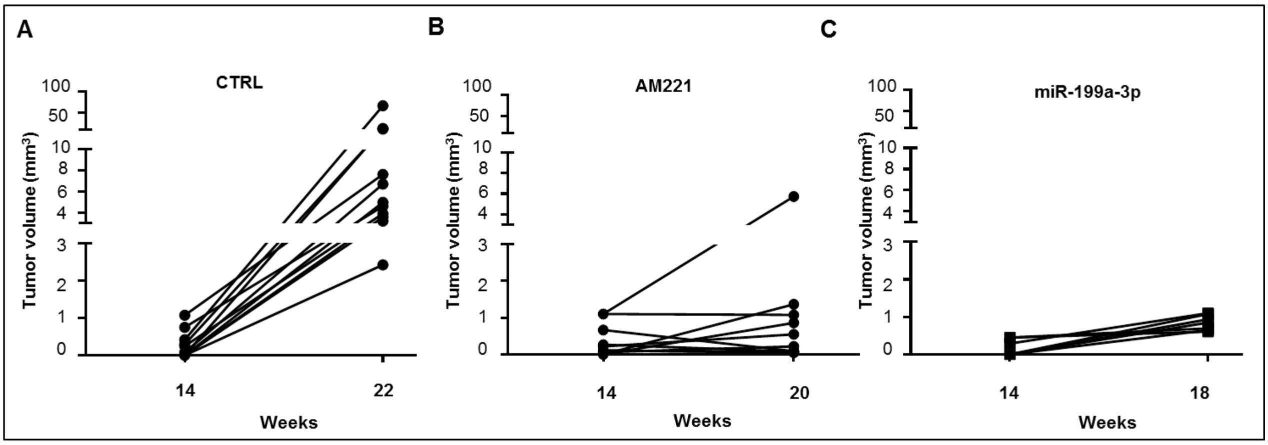

Figure S5. miRNA-based molecules induce a reduction in tumor growth. Mice were monitored by ultrasound to assess the development and growth of tumors during miRNA treatment. In control mice, the sustained growth of liver nodules was observed, whereas in miRNA-treated animals, the growth of tumor nodules was significantly delayed, which manifested as complete inhibition or regression in a few cases. Ultrasound analyses were performed at the following time points: 14 and 22 weeks for control animals (CTRL); 14 and 20 weeks for AM221-treated mice (AM221); 14 and 18 weeks for miR-199a-3p-treated mice (miR-199a-3p).

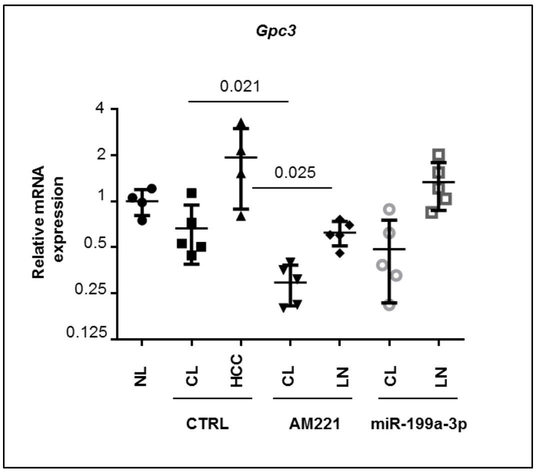

Figure S6. Reduction of *Gpc3* expression in miRNA-treated mice. Analysis of *Gpc3* mRNA by quantitative PCR in cirrhotic livers (CL) and liver nodules (LN) from TG221 (TG) mice treated with anti-miR-221 (AM221) or miR-199a-3p mimics. A significant decrease in mRNA levels of *Gpc3* was observed in AM221-treated animals, as compared to control (CTRL) mice (p values are reported on the plot). The vertical axis refers to mRNA expression normalized on normal livers

Table S1. Expression data of genes depicted in Figure 4B

GeneName				alpha fetoprotein	stearoyl-Coenzyme A desaturase 2	glypican 3	prominin 1	lipoprotein lipase	trefoil factor 3, intestinal	minichromosome maintenance deficient 2 mitotin (S. cerevisiae)	met proto-oncogene	yes-associated protein 1	macrophage stimulating 1 (hepatocyte growth factor-like)	CD44 antigen	hepatocyte growth factor	tumor-associated calcium signal transducer 2	vimentin	interleukin 6	bone morphogenetic protein 2
GeneSymbol				Afp	Scd2	Gpc3	Prom1	Lpl	Tff3	Mcm2	Met	Yap1	Mst1	Cd44	Hgf	Tacstd2	Vim	Il6	Bmp2
Genbank Accession				NM_007423	NM_009128	NM_016697	NM_008935	NM_008509	NM_011575	NM_008564	NM_008591	NM_001171147	NM_008243	NM_009851	NM_010427	NM_020047	NM_011701	NM_031168	NM_007553
Average NL TG				0.031	0.051	0.116	0.099	0.322	0.146	0.757	0.641	1.077	0.981	0.633	0.505	0.665	0.799	1.822	0.958
Average NL WT				0.024	0.050	0.095	0.097	0.152	0.013	0.777	0.535	0.930	1.078	0.488	0.534	0.722	0.509	0.946	0.940
Average CL TG				0.380	0.367	0.780	0.475	1.286	0.222	1.082	1.006	0.814	0.688	7.150	5.472	2.363	1.582	4.300	1.313
Average CL WT				0.024	0.166	0.484	0.882	0.780	0.020	0.492	0.989	0.812	0.801	1.764	4.233	1.390	1.758	0.739	1.420
Average HCC_CCl4 TG				0.971	1.461	1.107	1.422	2.724	2.665	1.654	0.753	0.652	0.507	4.194	4.208	2.071	2.541	3.920	0.920
Average HCC_DEN TG				10.113	3.989	22.016	3.241	2.583	2.010	1.240	1.221	1.160	1.411	1.269	0.475	1.223	1.406	1.788	0.836
Average HCC_DEN WT				6.803	3.557	12.272	3.472	1.762	0.972	1.904	0.786	1.146	1.226	0.431	0.530	0.657	1.314	1.199	0.778
**Genotype**	**Tumor**	**DEN**	**CCl_4**																
TG221	NL	no	no	0.049	0.057	0.084	0.105	0.523	0.052	1.040	0.571	0.957	0.925	0.671	0.406	0.572	0.357	0.904	0.740
TG221	NL	no	no	0.024	0.049	0.044	0.066	0.104	0.013	0.479	0.576	0.968	1.070	0.490	0.336	0.450	0.670	0.820	0.753
TG221	NL	no	no	0.034	0.076	0.216	0.105	0.148	0.006	0.952	0.783	0.843	0.988	0.667	0.842	0.697	1.201	1.631	1.015
TG221	NL	no	no	0.019	0.029	0.117	0.103	0.771	0.463	0.615	0.916	1.064	0.981	0.447	0.501	0.666	0.786	1.409	1.021
TG221	NL	no	no	0.033	0.035	0.148	0.109	0.274	0.335	0.662	0.615	1.543	0.989	0.945	0.547	0.741	1.073	5.207	0.583
TG221	NL	no	no	0.027	0.060	0.085	0.106	0.112	0.004	0.795	0.381	1.085	0.933	0.580	0.395	0.862	0.704	0.963	1.636
WT	NL	no	no	0.021	0.042	0.060	0.137	0.083	0.002	1.004	0.390	0.939	0.773	0.525	0.661	0.638	0.595	0.662	0.976
WT	NL	no	no	0.025	0.049	0.110	0.107	0.103	0.022	0.938	0.386	0.835	1.099	0.427	0.501	1.146	0.382	0.747	0.666
WT	NL	no	no	0.014	0.052	0.090	0.093	0.139	0.011	0.651	0.790	1.067	1.122	0.320	0.586	0.628	0.562	1.993	1.132
WT	NL	no	no	0.035	0.057	0.120	0.052	0.281	0.016	0.515	0.573	0.880	1.316	0.680	0.386	0.475	0.496	0.383	0.985
TG221	CL	no	yes	0.042	0.169	0.312	0.291	1.355	0.362	0.779	1.056	0.790	0.735	2.177	5.649	2.224	1.814	2.417	1.268
TG221	CL	no	yes	0.131	0.288	0.164	0.495	0.692	0.067	0.644	1.412	0.748	0.750	2.283	3.880	2.330	0.968	5.785	1.244
TG221	CL	no	yes	0.412	0.220	0.376	0.922	1.315	0.049	2.029	0.767	0.867	0.487	19.798	7.623	2.073	2.160	4.759	1.599
TG221	CL	no	yes	0.934	0.793	2.269	0.192	1.780	0.409	0.878	0.791	0.852	0.781	4.341	4.736	2.827	1.384	4.237	1.139
WT	CL	no	yes	0.032	0.179	0.626	1.274	0.921	0.020	0.447	1.068	0.842	0.696	2.321	4.927	1.103	2.393	0.956	1.155
WT	CL	no	yes	0.025	0.168	0.462	0.665	0.999	0.019	0.505	1.029	0.826	0.812	1.532	4.010	1.706	1.704	0.639	1.120
WT	CL	no	yes	0.022	0.171	0.381	0.627	0.589	0.018	0.589	0.897	0.806	0.839	1.487	4.333	1.967	1.363	0.609	1.610
WT	CL	no	yes	0.018	0.146	0.467	0.962	0.613	0.021	0.429	0.962	0.776	0.858	1.714	3.663	0.784	1.570	0.753	1.797
TG221	HCC	no	yes	1.259	2.847	0.925	1.040	2.441	5.284	1.056	0.605	0.655	0.457	3.616	3.883	1.084	1.496	2.794	1.118
TG221	HCC	no	yes	1.071	1.219	0.978	0.677	3.893	1.490	1.280	0.593	0.554	0.377	5.773	4.155	1.988	5.328	3.444	0.670
TG221	HCC	no	yes	0.816	1.155	0.429	2.832	2.885	3.555	3.411	0.884	0.525	0.474	3.420	3.644	3.311	1.749	6.859	0.877
TG221	HCC	no	yes	0.737	0.623	2.097	1.139	1.675	0.332	0.868	0.929	0.876	0.719	3.966	5.149	1.902	1.590	2.580	1.015
TG221	HCC	yes	no	4.912	4.183	7.007	1.253	2.742	2.306	1.189	0.597	1.124	1.117	2.251	0.652	1.099	2.254	1.817	0.612
TG221	HCC	yes	no	8.187	2.998	16.810	3.942	2.638	0.980	1.082	1.008	1.254	1.480	0.942	0.649	0.297	1.292	1.294	0.498
TG221	HCC	yes	no	6.564	3.767	39.873	4.296	1.684	2.675	1.831	1.589	0.981	1.260	1.394	0.480	0.546	1.203	2.205	0.713
TG221	HCC	yes	no	14.428	4.555	35.273	4.324	2.657	3.148	1.137	1.575	1.330	1.544	0.880	0.331	0.514	1.097	2.171	1.237
TG221	HCC	yes	no	11.252	2.887	30.332	3.195	3.666	1.717	0.875	1.353	1.163	1.529	1.058	0.321	0.932	1.369	2.569	1.042
TG221	HCC	yes	no	15.338	5.542	2.803	2.434	2.113	1.231	1.327	1.205	1.111	1.535	1.090	0.419	3.952	1.222	0.669	0.911
WT	HCC	yes	no	5.224	1.771	8.730	1.507	0.941	0.799	0.985	0.529	1.144	1.120	0.396	0.225	0.482	0.901	0.503	0.832
WT	HCC	yes	no	11.778	5.639	32.327	6.033	2.725	1.038	4.741	1.149	1.315	1.453	0.279	0.259	0.476	1.206	0.500	0.586
WT	HCC	yes	no	9.778	6.107	7.008	5.994	2.215	1.419	1.256	1.051	1.274	1.403	0.545	0.964	0.898	2.219	2.361	0.497
WT	HCC	yes	no	0.430	0.710	1.023	0.355	1.165	0.634	0.633	0.416	0.850	0.929	0.503	0.672	0.774	0.929	1.432	1.198

Table S2. Hystological characterization of miRNA-treated mice vs controls

	NODULES		HCC
	**< 500 µM**	**> 500 µM**	
**CONTROLS (n=9)**	66	24	4
**miR-199a-3p (n=6)**	44	12	0
**AM221 (n=7)**	33	10	1

Table S3. Sequences of primers used for PCR analysis

Gene Symbol	Gene name	mRNA	Forward Primer sequence	Reverse Primer sequence
**Afp**	alpha fetoprotein	NM_007423.4	GCACGAAAATGAGTTTGGGATAG	CTGGGTAAAGGTGATGGTAGC
**Tff3**	trefoil factor 3	NM_011575.2	CTGGGATAGCTGCAGATTACG	AGGGCACATTTGGGATACTG
**Scd2**	stearoyl-Coenzyme A desaturase 2	NM_009128.2	ATTTGGGAGCCTTGTACGG	CGTGCCTTGTATGTTCTGTG
**Lpl**	lipoprotein lipase	NM_008509.2	AACAAGGTCAGAGCCAAGAG	CCATCCTCAGTCCCAGAAAAG
**Gpc3**	glypican 3	NM_016697.3	AGAAACCTTATCCAGCCGAAG	AGTTCTTGTCCGTTCCAGC
**Tgfb1**	transforming growth factor, beta 1	NM_011577.2	CCTGAGTGGCTGTCTTTTGA	CGTGGAGTTTGTTATCTTTGCTG
**Ctgf**	connective tissue growth factor	NM_010217.2	CTCCACCCGAGTTACCAATG	TGGCGATTTTAGGTGTCCG
**α-Sma**	alpha smooth muscle actin	NM_007392.3	GTGAAGAGGAAGACAGCACAG	GCCCATTCCAACCATTACTCC
